# Shank2 Regulates Renal Albumin Endocytosis

**DOI:** 10.14814/phy2.12510

**Published:** 2015-09-02

**Authors:** Evgenia Dobrinskikh, Linda Lewis, R Brian Doctor, Kayo Okamura, Min Goo Lee, Christopher Altmann, Sarah Faubel, Jeffrey B Kopp, Judith Blaine

**Affiliations:** 1 University of Colorado Anschutz Medical CampusAurora, Colorado; 2 University of MississippiOxford, Mississippi; 3 Department of Pharmacology, Severance Biomedical Science Institute, Yonsei University College of MedicineSeoul, Korea; 4 Kidney Disease Section, National Institute of Diabetes and Digestive and Kidney Disease, National Institutes of HealthBethesda, Maryland

**Keywords:** Advanced imaging, albuminuria, endocytosis, scaffolding proteins

## Abstract

Albuminuria is a strong and independent predictor of kidney disease progression but the mechanisms of albumin handling by the kidney remain to be fully defined. Previous studies have shown that podocytes endocytose albumin. Here we demonstrate that Shank2, a large scaffolding protein originally identified at the neuronal postsynaptic density, is expressed in podocytes in vivo and in vitro and plays an important role in albumin endocytosis in podocytes. Knockdown of Shank2 in cultured human podocytes decreased albumin uptake, but the decrease was not statistically significant likely due to residual Shank2 still present in the knockdown podocytes. Complete knockout of Shank2 in podocytes significantly diminished albumin uptake in vitro. Shank2 knockout mice develop proteinuria by 8 weeks of age. To examine albumin handling in vivo in wild-type and Shank2 knockout mice we used multiphoton intravital imaging. While FITC-labeled albumin was rapidly seen in the renal tubules of wild-type mice after injection, little albumin was seen in the tubules of Shank2 knockout mice indicating dysregulated renal albumin trafficking in the Shank2 knockouts. We have previously found that caveolin-1 is required for albumin endocytosis in cultured podocytes. Shank2 knockout mice had significantly decreased expression and altered localization of caveolin-1 in podocytes suggesting that disruption of albumin endocytosis in Shank2 knockouts is mediated via caveolin-1. In summary, we have identified Shank2 as another component of the albumin endocytic pathway in podocytes.

## Introduction

Albuminuria is strongly associated with kidney disease progression (Hemmelgarn et al. 2010; Hallan et al. 2012). Given the clinical significance of albuminuria as a biomarker, the mechanisms involved in the development of albuminuria are an area of active investigation. Renal handling of albumin is complex and both the glomerulus and the proximal tubule have been shown to be involved in albumin handling (Eyre et al. 2007; Russo et al. 2007; Sandoval et al. 2012; Dobrinskikh et al. 2014).

The structure that impedes the passage of albumin from the blood to the urinary ultrafiltrate is the glomerular filtration barrier (GFB). The GFB consists of three adjacent layers – fenestrated vascular endothelial cells, the glomerular basement membrane, and podocytes. Podocytes are specialized epithelial cells which have a large cell body and multiple processes that extend to cover the glomerular capillaries. Processes that ramify from the podocyte cell body divide into progressively smaller processes to finally form foot processes which abut the glomerular basement membrane and interdigitate with neighboring podocytes. Paracellular passage of molecules between podocytes is restricted by the slit diaphragm which contains integral membrane proteins that extend between foot processes of adjacent podocytes. Under normal conditions, passage of albumin through the filtration barrier could occur via paracellular flux across the slit diaphragm or via transcellular uptake through the podocyte. In support of the uptake and passage of albumin through the podocyte are studies showing that cultured podocytes endocytose and transcytose albumin (Eyre et al. 2007; Dobrinskikh et al. 2014) and that in proteinuric kidney diseases albumin is found in podocytes in native glomeruli (Eddy et al. 2000; Tojo et al. 2008; Kinugasa et al. 2011; Tojo and Kinugasa 2012).

While the precise amount of albumin that passes through the GFB is a matter of active debate, by even the most conservative estimates between 1 and 3 g a day of albumin passes through the GFB (Tojo and Endou 1992; Tojo and Kinugasa 2012). In diseases causing proteinuria, the GFB becomes leaky allowing the passage of significant amounts of albumin into the urine. Albumin that passes through the intact GFB under normal conditions or the damaged filtration barrier in disease states is taken up by renal proximal tubular cells (Tojo et al. 2001; Russo et al. 2007). The mechanisms involved in albumin uptake and disposal within the glomerulus and the proximal tubule remain to be fully defined.

The Shank family of proteins are a family of large scaffolding proteins containing three known members: Shank1, Shank2, and Shank3 (Sheng and Kim 2000). Shank2 is a scaffolding protein originally discovered at the neuronal postsynaptic density where it regulates the movement of proteins to and from the synaptic membrane adjacent to the synaptic cleft (Naisbitt et al. 1999; Sheng and Kim 2000). Shank2 binds proteins involved in modulating actin dynamics and endocytosis regulation including dynamin2 and cortactin (Du et al. 1998; Okamoto et al. 2001). Shank2 is expressed in epithelial cells including renal tubular cells (Dobrinskikh et al. 2010; Jung et al. 2014). In previous work, we have found that Shank2 plays a pivotal role in endocytosis of the sodium phosphate cotransporter 2a (NaPi2a) in the renal proximal tubule (Dobrinskikh et al. 2010). Here, using Shank2 knockout mice and cultured podocytes we examine the role of Shank2 in albumin endocytosis in podocytes and renal proximal tubule cells. We find that podocytes, in addition to renal proximal tubular cells, express Shank2 and that Shank2 is required for albumin uptake. These studies further define the mechanisms underlying the renal handling of albumin and may ultimately lead to therapies to slow the progression of proteinuric kidney disease.

## Materials and Methods

### Animals

Male Sprague Dawley rats (200–250 g) were obtained from Harlan Laboratories (Madison, WI). Shank2 knockout mice have been described previously (Won et al. 2012). Animals were housed at the University of Colorado Anschutz Campus Animal Facility and had free access to food and water. All procedures were performed in accordance with protocols approved by the Institutional Animal Care and Use Committee, University of Colorado Denver. To assess proteinuria, the amount of protein in urine samples from wild-type or knockout mice was measured using the Albuwell kit (Exocell, Sigma). Urinary creatinine was measured using the QuantiChrom Creatinine Assay Kit from BioAssay Systems (Hayward, CA).

### Cell culture

#### Human urine derived podocytes

Human urine derived podocyte-like epithelial cells (HUPEC) carrying genes encoding a temperature-sensitive variant of the simian virus (SV40) large tumor antigen and human telomerase were cultured and maintained, as previously described (Sakairi et al. 2010). Briefly, podocytes were grown on type I collagen-coated flasks (Fisher Scientific, Rochester, NY) at 33°C (growth-permissive conditions) in growth media (RPMI 1640 medium [Sigma, St. Louis, Missouri], 10% fetal bovine serum [Hyclone, Logan, UT], 15 mmol/L HEPES, 5 mmol/L l-glutamine, 100 U/mL penicillin, 100 U/mL streptomycin, 5% CO_2_ atmosphere). After reaching confluence, cells were seeded onto 35-mm type I collagen-coated dishes and maintained at 37°C (growth-restrictive conditions) for 7–12 days to allow for differentiation. All cells were used between passages 17 and 25.

#### Primary mouse podocytes

Glomeruli were isolated from 8- to 10-week-old male wild-type (litter mate) or Shank2^−/−^ (Shank2 knockout) mice as described previously (Assmann et al. 1991). Cells that grew out from the isolated glomeruli were subsequently trypsinized between days 5 and 7 and passed through a 25-*μ*m filter to remove mesangial cells as described previously (Harper et al. 1984). After confluency was obtained, cells were stained for podocin and WT-1 to confirm that they were podocytes. More than 98% of cells were positive for podocin and WT-1. Primary podocytes were not used for more than four passages from initial isolation.

### Immunofluorescence imaging of rat renal podocytes

Male Sprague Dawley rats were used to immunolocalize specific proteins within renal tissues, as described previously (Doctor et al. 1993). For the immunolocalization studies, the kidneys were cleared of blood by cannulating the descending aorta and retrograde perfusion of phosphate-buffered saline (PBS) and then fixed by perfusion with 4% paraformaldehyde (Electron Microscopy Sciences; Hatfield, PA) in PBS (pH 7.4). The kidneys were then removed, immersed in 4% paraformaldehyde for 60 min, cut into 3–4 mm cubes, infused with 5% (2 h), 10% (2 h), and 25% (overnight) sucrose, frozen in liquid nitrogen, and cryosectioned (5–8 *μ*m). Kidney sections were blocked (10% normal goat serum and 1% bovine serum albumin [BSA] in PBS) and incubated overnight at 4°C with primary antibody against Rb polyclonal Shank2 (1:100, Santa Cruz; Santa Cruz, CA). After washing, the sections were incubated (30 min, room temperature) with a mix of Alexa 488-conjugated goat anti-rabbit IgG (1:500; Invitrogen, Carlsbad, CA), Alexa 546-conjugated phalloidin (1:100; Invitrogen, Carlsbad, CA). Sections were then washed with PBS and mounted in Mowiol 4-88 (Calbiochem, Gibbstown, NJ) containing 2.5% 1,4-diazabicyclo[2.2.2]octane (Sigma, St. Louis, MO). Fluorescence images were obtained using Zeiss Axiovert 200M laser-scanning confocal/multiphoton-excitation fluorescence microscope with a Meta spectral detection system (Zeiss NLO 510 with META, Zeiss, Thornwood, NY). The imaging settings were initially defined empirically to maximize the signal-to-noise ratio and to avoid saturation. In comparative imaging, the settings were kept constant between samples. Series of confocal fluorescence images were simultaneously obtained with a Zeiss C-Apochromat 40×/1.2NA water-immersion lens objective. The illumination for imaging was provided by a 30 mW Argon Laser, HeNe 5 mW (633 nm), and 1 mW (543 nm). Image processing was performed using Zeiss ZEN 2009 software. Figures were mounted using Adobe Photoshop CS4 (Adobe System Inc.)

### FITC-albumin uptake/degradation/transcytosis

FITC-labeled albumin was used to measure uptake by cultured podocytes. Two hours prior to the experimental procedures, the media was changed to Ringer solution (122.5 mmol/L NaCl, 5.4 mmol/L KCl, 1.2 mmol/L CaCl_2_, 0.8 mmol/L MgCl_2_, 0.8 mmol/L Na_2_HPO_4_, 0.2 mmol/L NaH_2_PO_4_, 5.5 mmol/L glucose, and 10 mmol/L HEPES; pH 7.4). For albumin uptake experiments, podocytes were incubated with 1.5 mg/mL human FITC-albumin (MP Biomedicals, Santa Ana, CA) in Ringer solution at either 4°C or 37°C for 0, 60, and 120 min. For the 4°C condition, cells were kept at 4°C for the duration of the experiment. After the specified times, cells were washed six times with ice-cold PBS and then lysed in ice-cold RIPA buffer (150 mmol/L sodium chloride, 1.0% NP-40 or Triton X-100, 5% sodium deoxycholate, 0.1% sodium dodecyl sulfate, 50 mmol/L Tris, pH 8.0).

### Western blotting

For podocytes lysed in RIPA buffer, protein concentrations were measured by BCA assay (Pierce; Rockford, IL) and samples were reduced (10% *β*-mercaptoethanol). Cell lysates were run on 10% polyacrylamide gels and transferred onto nitrocellulose membranes (Bio-Rad, Hercules, CA). Subsequent blocking, antibody, and wash solutions were diluted in blot buffer (150 mmol/L NaCl, 10 mmol/L Na_2_HPO_4_, 5 mmol/L EDTA, 1% Triton X-100; pH 7.4). Membranes were initially blocked (5% nonfat dry milk; 60 min) and then incubated with primary antibody against FITC (1:1000; clone ZF2471-1900, Invitrogen, Carlsbad, CA) and GAPDH (1:1000; FL-335, Santa Cruz Biotech; Santa Cruz, CA). Blots were then washed, incubated with horseradish peroxidase-conjugated secondary antibodies (1:10,000 dilution; Jackson ImmunoResearch, West Grove, PA), and washed. The antibody complexes were detected using enhanced chemiluminescence (Pierce; Rockford, IL) and captured using a photodocumentation system (UVP; Upland, CA).

### Immunofluorescence

Confocal microscopy images were acquired using Zeiss Axiovert 200M laser-scanning confocal/multiphoton-excitation fluorescence microscope with a Meta spectral detection system (Zeiss NLO 510 with META, Zeiss, Thornwood, NY). The imaging settings were initially defined empirically to maximize the signal-to-noise ratio and to avoid saturation. In comparative imaging, the settings were kept constant between samples. Series of confocal fluorescence images were simultaneously obtained with a Zeiss C-Apochromat Zeiss Plan-Apochromat 63/1.4NA oil objective. The illumination for imaging was provided by a 30 mW Argon Laser, HeNe 5 mW (633 nm), and 1 mW (543 nm). Image processing was performed using Zeiss ZEN 2009 software. Figures were mounted using Adobe Photoshop CS4 (Adobe System Inc.)

For fixed cell images, podocytes were incubated in 4% paraformaldehyde in phosphate-buffered saline (PBS) with 0.5% Triton X-100 (20 min; room temperature), washed, blocked with 10% normal serum, and labeled with primary antibodies. Primary antibodies include: Shank2 (1:100, Santa Cruz; Santa Cruz, CA), EEA1 (1:100; 14/EEA1, Becton-Dickinson; Franklin Lakes, NJ), LAMP1 (1:100; LY1C6, Enzo, Farmingdale, NY), caveolin-1 (1:100; ab2910, Abcam, Cambridge, MA). Cells were subsequently washed and labeled with the appropriate conjugated secondary antibodies (Alexa Fluor 488, Alexa Fluor 633; Invitrogen, Carlsbad, CA). F-actin was concurrently stained with Alexa-Phalloidin 633 or Alexa-Phalloidin 546 (Invitrogen; Carlsbad, CA).

The degree of colocalization of FITC-albumin with different markers within the cells was quantified by calculating their intensity correlation quotient (ICQ) values (Li et al. 2004; Dobrinskikh et al. 2010). Intensity_Correlation_Analysis macros (www.macbiophotonics.ca) for this analysis were applied to Image J (National Institute of Mental Health, Bethesda, MD). Values for the product of the differences from the mean (PDM) quantify the degree of pixel-by-pixel synchrony of the fluorophore intensities in the two channels [PDM = (*R*_i_ − *R*_ave_) × (*G*_i_ − *G*_ave_); *R*_i_ = individual pixel intensity-red, *R*_ave_ = average pixel intensity-red, *G*_i_ = individual pixel intensity-green, *G*_ave_ = average pixel intensity-green]. The ICQ (intensity correlation quotient) ratio is equal to the ratio of the number of positive PDM values to the total number of pixel values (ICQ = (*N*_+ve_/*N*_total_); *N*_+ve_ = number of pixels with a positive PDM value, *N*_total_ = total number of pixels with a non-zero value in each channel). The ICQ values are distributed between −0.5 and +0.5 by subtracting 0.5 from this ratio. A positive ICQ value implies colocalization, whereas a negative ICQ value implies no colocalization. Eleven focal planes of 10 individual podocytes for each marker after 0 and 1 h of FITC-albumin uptake were analyzed (Image J; NIH, Bethesda, MD).

### Transmission electron microscopy

Transmission electron microscopy was performed at the Electron Microscopy Core at the University of Colorado Denver. For the these studies, the kidneys from 8-week-old Shank2 wild-type and knockout mice were cleared of blood by perfusion through the heart with phosphate-buffered saline (PBS) and then fixed by perfusion with 2% glutaraldehyde (Electron Microscopy Sciences; Hatfield, PA) in 0.1 mol/L cacodylate buffer. The kidneys were then removed, cut into 1–2 mm cubes, fixed in 2% glutaraldehyde overnight, postfixed in 1% osmium tetroxide in 0.1 mol/L cacodylate buffer for 1 h, dehydrated in an ethanol series for 15 min each (50%, 70%, 95%, and two changes of 100% ethanol), embedded into Embed 812 resin and baked at 60°C for 48 h. Thick sections (0.5–1.0 *μ*m) were cut and stain with toluidine blue stain for 2–5 min for precise ultrathin sectioning. Ultrathin sections (60–90 nm thick) were cut and collected onto grids. Grids were stained with uranyl acetate and lead citrate prior to imaging with a Technai G2 electron microscope (FEI) using a Gatan FirstLight camera. Figures were mounted using Adobe Photoshop CS4 (Adobe System Inc.)

### Shank2 knockdown

For the Shank2 knockdown lentiviral particles encoding short-hairpin RNA against Shank2 (MISSION TRC shRNA, Sigma-Aldrich, St. Louis, MO) were transduced into HUPEC podocytes using the company recommended protocol.

### Intravital imaging

Intravital imaging was performed as described previously (Schiessl et al. 2013). Briefly, 10- to 16-week-old Shank2 wild-type or knockout mice were anesthetized and a jugular venous catheter and endotracheal tube were inserted. The left kidney was exteriorized and placed glass-bottom MatTek dish (MatTek Corporation, Ashland, MA) mounted in a custom-made dish holder. Images were obtained using Zeiss 780 laser-scanning confocal/multiphoton-excitation fluorescence microscope with a 34-Channel GaAsP QUASAR Detection Unit and nondescanned detectors for two-photon fluorescence (Zeiss, Thornwood, NY). The imaging settings were initially defined empirically to maximize the signal-to-noise ratio and to avoid saturation. In comparative imaging, the settings were kept constant between samples. Time-series of confocal images were obtained with a Zeiss C-Apochromat 40x/1.2NA Korr FCS M27 water-immersion lens objective. The illumination for imaging was provided by a tunable infrared Coherent Chameleon Ultra II laser (680–1080 nm). Image processing was performed using Zeiss ZEN 2012 software. Figures were mounted using Adobe Photoshop CS4 (Adobe System Inc.) Nuclei were visualized by injection of 25 *μ*L of a 10 mg/mL solution of Hoechst dye (Invitrogen). Albumin trafficking was examined by injection of 50 *μ*L of a 20 mg/mL solution of FITC-bovine serum albumin (Sigma). Images were acquired every 2 sec over 20 min.

### Statistical analysis

All data are presented as mean ± SEM. Statistical analysis was performed using *t*-tests for pairs of data and one-way analysis of variance (ANOVA) for data groups of three or more using Prism-4 GraphPad software. Tukey's post hoc test was applied to the ANOVA data. Values were considered statistically significant when *P* < 0.05.

## Results

### Shank2 is expressed in podocytes and proximal tubules

**Figure 1:**
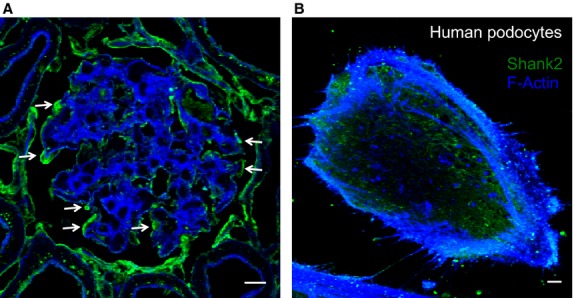
Shank2 is expressed in podocytes in vivo and in vitro. (A) Immunofluorescence of Shank2 (green) in podocytes in rat glomerulus (arrows). Actin is stained blue. Bar – 10 *μ*m. (B) Immunofluorescence of Shank2 (green) in human podocytes. Actin is stained blue. We are unable to demonstrate Shank2 staining using IF in mouse podocytes because our Shank2 antibody does not work for IF on mouse tissue (but does work for western blot, see Fig. 5B). Bar – 10 *μ*m.

A first step in determining whether Shank2 is involved in albumin endocytosis in the kidney is evaluating Shank2 renal expression. In previous studies, we have shown that Shank2 is expressed in the proximal tubule (Dobrinskikh et al. 2010). Here, we demonstrate that Shank2 is expressed in podocytes in vivo and in vitro. Figure 1 demonstrates staining for Shank2 in glomerular podocytes (Fig. 1A, arrows) as well as in cultured podocytes (Fig. 1B).

### Shank2 colocalizes with albumin in podocytes

**Figure 2:**
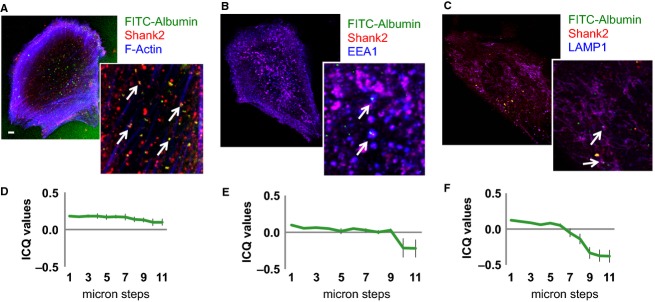
Association of albumin-laden endosomes, distinct endosomal compartments, and Shank2. (A–C) Podocytes were treated with FITC-albumin (*green*; 10 min; 37°C), fixed and stained for Shank2 (r*ed*) with (B) EEA1 (*blue*), (C) LAMP1 (*blue*), and F-Actin (*blue*). FITC-albumin partially colocalizes (*arrows*) with early endosomes and lysosomes. Shank2 also colocalizes (*arrows*) with FITC-albumin containing endosomes. Bar – 10 *μ*m, the same magnification was used for all images. (D–F) Intensity correlation coefficient analysis indicates there is a spatial dependency on the degree of colocalization of albumin with early endosomes and lysosomes, but not Shank2. Graph abscissas denote micron steps above the coverslip level. (D) Shank2 colocalization remained constant in all levels of the podocytes, but for (E) EEA1 and (F) LAMP1, colocalization diminished at the upper reaches of the podocytes.

To further delineate the role of Shank2 in albumin trafficking pathways in podocytes we examined whether albumin colocalizes with Shank2 after internalization. Human podocytes were treated with fluorescein isothyocyanate (FITC)-labeled albumin for 10 min, washed, and then stained for Shank2, the early endosomal marker EEA1, or the lysosomal marker LAMP1. Optical sections (z-stacks) from the bottom of the podocytes to the top were obtained to examine whether there were differences in albumin distribution between the basal and apical aspects of the podocyte. ICQ values for each section (from the bottom of the podocyte to the top) were calculated to assess the degree of colocalization between Shank2 and FITC-albumin (Fig. 2D), Shank2 and EEA1 (Fig. 2E), and Shank2 and LAMP1 (Fig. 2F). After 10 min of incubation with FITC-albumin, Shank2 was seen to colocalize with FITC-albumin throughout the podocyte (Fig. 2A and D). In contrast, FITC-albumin preferentially colocalized with the early endosomal marker EEA1 or the lysosomal marker LAMP1 at the basal aspect of the podocyte (Fig. 2B, C, E, F). This suggests that Shank2 accompanies FITC-albumin throughout its passage through the podocyte.

### Lack of Shank2 impairs albumin endocytosis in podocytes

**Figure 3:**
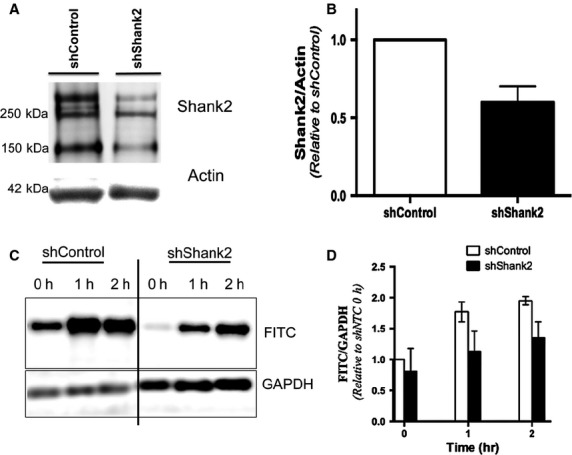
Shank2 knockdown decreases albumin uptake in podocytes. (A) Western blot analysis of cultured human podocytes infected with nontarget control or Shank2 shRNA. Shank2 gives bands at ˜250 kD and 150 kD. (B) Densitometric analysis of Shank2 knockdown using lentiviral shRNA. Shank2 expression is significantly reduced to 46 ± 16% of control levels in podocytes infected with Shank2 shRNA (*P* < 0.01) *P* value to figure. (C) Western blot analysis of FITC-albumin uptake in control (shControl) versus Shank2 knockdown (shShank2) podocytes. (D) Densitometric analysis of FITC-albumin uptake in control (shControl) versus Shank2 knockdown podocytes (shShank2). Intensities were normalized to shNTC 0 h condition. Shank2 knockdown decreased FITC-albumin uptake by 36 ± 23% compared to nontarget control (*P* < 0.1) after 1 h of uptake and by 40 ± 24% compared to nontarget control after 2 h of uptake (*P* < 0.07), although as noted these decreases did not reach statistical significance (*n *=* *3 experiments).

To examine whether Shank2 is required for albumin uptake in podocytes, we knocked down Shank2 in human podocytes and examined FITC-albumin uptake. Knockdown of Shank2 using lentiviral shRNA resulted in a 64 ± 16% reduction in Shank2 expression compared to nontarget control (Fig. 3A and B). Shank2 knockdown decreased FITC-albumin uptake by 36 ± 23% compared to nontarget control (*P* < 0.1) after 1 h of uptake and by 40 ± 24% compared to nontarget control after 2 h of uptake (*P* < 0.07) (Fig. 3C and D). While inhibition of albumin uptake in Shank2 knockout podocytes was almost significant after 2 h of FITC-albumin uptake, failure to achieve a significant decrease in albumin uptake was likely due to the residual expression of Shank2.

**Figure 4:**
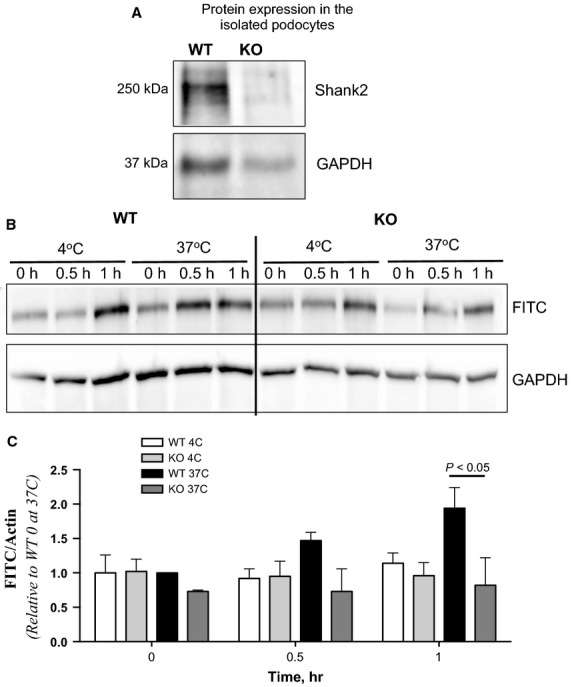
Shank2 knockout inhibits albumin uptake in podocytes. (A) Podocytes isolated from Shank2 knockout mice do not express Shank2. (B) Representative western blot of lysates from wild-type or Shank2 knockout podocytes incubated with FITC-albumin at 4°C (inhibits endocytosis, permits binding) or 37°C (permits endocytosis) for the times shown. (C) Densitometric analysis of the amount of FITC-albumin present in wild-type or Shank2 knockout podocyte cellular lysates. Intensities were normalized to the intensity at 37°C, 0 h for wild-type or Shank2 knockout podocytes. *P < 0.05, *n* = 3 independent experiments.

Since Shank2 is a relatively large protein (˜250 kD), it is difficult to completely knock it down in podocytes. We thus examined FITC-albumin uptake in primary podocytes isolated from Shank2 knockout mice. Podocytes isolated from Shank2 knockout mice do not express Shank2 (Fig. 4A). Control or Shank2 knockout podocytes were incubated with FITC-albumin at 4°C (inhibits albumin endocytosis and allows assessment of albumin binding) and 37°C (permits endocytosis). FITC-albumin uptake after 60-min incubation at 37°C was significantly decreased in Shank2 knockout podocytes compared to wild type (1.94 ± 0.30 versus 0.82 ± 0.40; *P* < 0.05), indicating that Shank2 plays an important role in albumin uptake in podocytes (Fig. 4B and C).

### Albumin trafficking in vivo differs between wild-type and Shank2 KO mice

**Figure 5:**
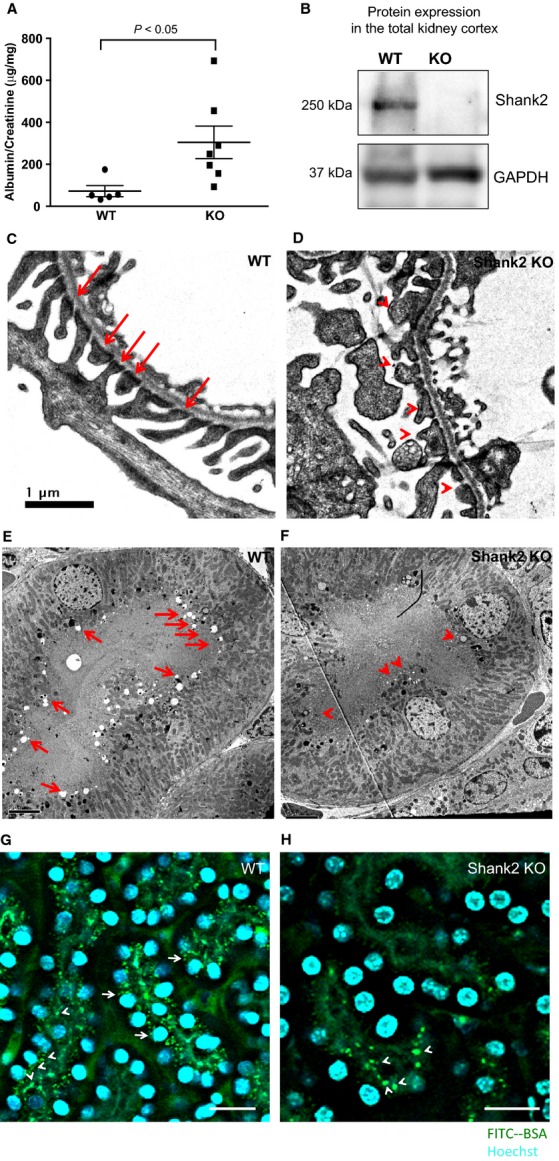
Albumin trafficking is impaired in Shank2 knockout mice in vivo. (A) Urinary albumin normalized to creatinine in wild-type (*n *=* *5) and Shank2 KO mice (*n *=* *7). (B) Shank2 knockout mice have no Shank2 expression in the renal cortex. (C) Transmission electron microscopy (TEM) demonstrates normal foot processes in wild-type mice (arrows). (D) In contrast, TEM shows broadening of podocyte foot processes in Shank2 KO and mild podocyte foot process effacement (arrowheads). (E) TEM demonstrates abundant endocytotic vesicles at the apical surface of a proximal tubule cell in a wild-type mouse (arrows). (F) There are significantly fewer vesicles (arrowheads) at the apical proximal tubular membrane of a Shank2 knockout mouse. (G) Intravital multiphoton images taken 15 min after FITC-albumin injection demonstrate FITC-albumin below the brush border in renal tubular cells in a wild-type mouse (arrowheads). In some cells, internalized FITC-albumin is localized close to the basal aspect of the cell (arrows). (H) In contrast, 15 min after FITC-albumin injection into a Shank2 knockout mouse, the majority of renal tubules contain no albumin. In a few cells, the injected FITC-albumin appears stuck on the brush border where it aggregates in large vesicles (Fig. 5H, arrowheads). Bar – 20 *μ*m.

Shank2 knockout mice develop albuminuria by 8 weeks of age (Fig. 5A) and have no Shank2 expression in the renal cortex (Fig. 5B). Histologic analysis of podocytes using transmission electron microscopy (TEM) shows broadening of podocyte foot processes in Shank2 KO compared to wild type (Fig. 5D vs. 5C) and mild podocyte foot process effacement. TEM analysis of proximal tubules demonstrates a marked reduction in endocytic vesicles in the Shank2 knockout compared to wild type (Fig. 5F vs. 5E). Thus, albuminuria in the Shank2 knockout appears to be a combination of albumin leaking through a damaged filtration barrier and decreased proximal tubular uptake of albumin.

To study renal protein trafficking in Shank2 knockout mice in a mechanistic fashion, we examined albumin handling in vivo using multiphoton imaging, a dynamic imaging technique that allows visualization of in vivo processes with minimal phototoxicity (Dunn et al. 2012; Schiessl and Castrop 2013). Injection of FITC-albumin into wild-type mice resulted in the rapid uptake of albumin into renal tubules (Fig. 5G, Video S1 and S3). The FITC-albumin was internalized and then moved toward the basal aspect of the tubular cells (Fig. 5G, arrows; Video S3). In contrast, after injection of FITC-albumin into Shank2 knockout mice, the majority of tubules showed no albumin uptake (Fig. 5H; Video S2 and S4). In a few tubules, the albumin accumulated in large vesicles which stuck to the brush border and were not internalized (Fig. 5H, arrowheads). The dramatic decrease in albumin uptake in knockout mice compared to wild-type was consistently seen in all groups of mice examined (*n *=* *3 WT and *n *=* *4 KO). Regional blood flow to the kidneys was equal in wild-type and Shank2 knockout mice as evidenced by the fact that in both mice the uptake of Hoechst dye which stains nuclei occurred very rapidly after injection of the dye. Taken together, data from the in vitro albumin trafficking, TEM, and intravital experiments suggest that albuminuria in Shank2 knockout mice is a combination of albumin leaking through a damaged filtration barrier (bypassing the normal endocytotic mechanisms in podocytes for handling albumin) and decreased proximal tubular uptake of albumin.

### Caveolin-1 expression is altered in Shank2 knockout mice

**Figure 6:**
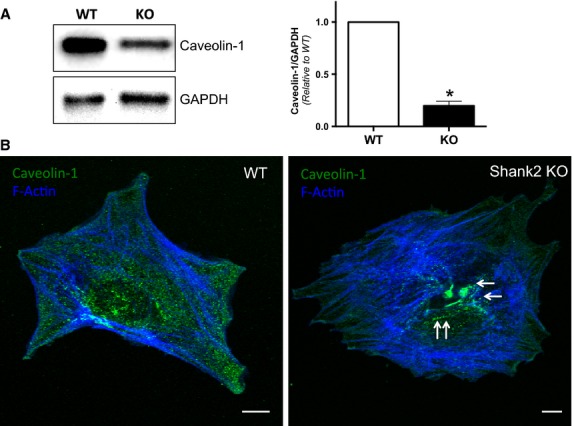
Caveolin-1 expression is altered in Shank2 knockout podocytes. (A) Western blot analysis demonstrates decreased caveolin-1 expression in Shank2 knockout podocytes compared to wild type. Densitometric analysis indicates a 70 ± 6% reduction in caveolin expression in the knockout podocytes (*P < 0.05, *n* = 3 experiments). (B) Representative immunofluorescence images demonstrate that caveolin-1 is mislocalized away from the cell edge in Shank2 knockout podocytes compared to wild type. Bar – 10 *μ*m.

In previous work, we have found that albumin uptake in podocytes is mediated by caveolin-1 (Dobrinskikh et al. 2014). Since Shank2 is a scaffolding protein, it may be involved in modulating the expression of caveolin-1. In both cultured podocytes in vitro and in vivo, we found that caveolin-1 expression was decreased in the knockout mice (Fig. 6A and B). In addition, caveolin-1 expression in cultured podocytes was mislocalized from the cell membrane toward the interior of the cell (Fig. 6B, arrows). Thus, one of the mechanisms whereby albumin uptake is impaired in Shank2 knockout podocytes may be through mislocalization and decreased expression of caveolin-1.

## Discussion

Albuminuria is one of the hallmarks of kidney disease and thus there is intense interest in determining precisely how the kidney handles albumin under physiologic and disease conditions. Traditionally, only a small amount of serum albumin was thought to traverse the GFB and enter the urinary space. This model has recently been challenged with in vivo multiphoton imaging studies showing nephrotic levels of albumin traversing the GFB in normal animals (Russo et al. 2007; Sandoval et al. 2012). Albumin has also been found within the podocyte cell body in human and rodent proteinuric kidney disease (Tojo et al. 2008; Kinugasa et al. 2011; Tojo and Kinugasa 2012). In vivo multiphoton imaging of rat renal glomeruli found that the glomerular sieving coefficient for albumin (GSC_A_) varies from ˜0.016 to ˜0.034 (Russo et al. 2007; Sandoval et al. 2012). This is much higher than that obtained by traditional micropuncture techniques which gave a GSC_A_ of 0.00062 in rats (Tojo and Endou 1992). Depending on the value used to calculate the GSC_A_ (0.00062–0.034), the amount of albumin filtered through the glomerulus could range from 3 g to 200 g per day.

While several theories have been proposed to explain why the GFB does not clog with abundant serum proteins such as albumin and IgG (Smithies 2003), podocyte uptake and disposal of serum proteins provides a biologically plausible mechanism for maintaining GFB homeostasis. Evidence that podocytes play a central role in preventing GFB clogging comes from the following observations. In animals with heavy proteinuria, albumin-filled vesicles are found within the podocyte cell body (Tojo et al. 2008; Kinugasa et al. 2011), podocytes have been shown to endocytose albumin in vitro (Eyre et al. 2007; Dobrinskikh et al. 2014), IgG appears to collect in the GFB in mice that lack the neonatal Fc receptor, a protein required for transcytosis (Akilesh et al. 2008), and cultured podocytes possess both transcytotic and degradative pathways for clearing albumin (Dobrinskikh et al. 2014).

Albumin that is transcytosed through the GFB is taken up by renal proximal tubules (Tojo et al. 2001; Russo et al. 2007; Tojo and Kinugasa 2012). In disease states, damage to the GFB and to the proximal tubules leads to both an increase in albumin filtration through the GFB and saturation of the mechanisms of tubular albumin uptake resulting in albuminuria (Tojo et al. 2001, 2008; Vinge et al. 2010). The mechanisms whereby podocytes and renal proximal tubules handle albumin remain to be fully defined. Previous work has shown that Shank2 is expressed in the proximal tubule but other sites of renal expression had not been defined. The present studies show that Shank2 is expressed in both podocytes and renal tubular cells and that Shank2 regulates the uptake of albumin both in vivo and in vitro.

Shank2 is a large scaffolding protein originally identified as a key component of the neuronal postsynaptic density (Naisbitt et al. 1999). Shank proteins contain a multitude of protein–protein binding sites including ankyrin repeats, an SH3 domain, a PDZ domain, proline-rich repeats and a SAM domain. The full-length Shank protein can also multimerize (Naisbitt et al. 1999), promoting the formation of large complexes of proteins. Shank proteins have been shown to bind to a number of different proteins involved in signaling and regulation of the actin cytoskeleton including cortactin (Du et al. 1998), dynamin2 (Okamoto et al. 2001), and *β*Pix – a guanine nucleotide exchange factor for Rho GTPase (Lee et al. 2010). In addition, Shank2 has been shown to be involved in trafficking of the sodium phosphate cotransporter NaPi2a in renal proximal tubule cells (Dobrinskikh et al. 2010) and in regulating the activity of the cystic fibrosis transmembrane conductance regulator in intestinal epithelial cells (Jung et al. 2014). Since Shank2 interacts with several proteins involved in regulating endocytosis and actin cytoskeleton dynamics, the role of Shank2 in albumin uptake in podocytes was investigated. Reduced expression of Shank2 impaired albumin endocytosis in cultured podocytes. Multiphoton intravital imaging of the kidney cortex in wild-type and Shank2 knockout mice found significant differences in albumin endocytosis. While Shank2 wild-type mice showed rapid (within one minute of albumin injection) uptake of injected FITC-albumin into renal tubular cells, there was minimal uptake of injected FITC-albumin in the Shank2 knockout. In wild-type mice, endocytosed albumin entered the renal tubular cell at the apical aspect and then was trafficked toward the basal cell surface. In contrast, in the knockout mice, in the few tubular cells in which FITC-albumin was seen, albumin appeared to bind to the apical brush border but then failed to be further internalized, suggesting a defect in albumin trafficking in Shank2 knockout renal tubular cells. The differences in albumin uptake between wild-type and knockout mice were not due to differences in regional blood flow as, after intrajugular injection, Hoechst dye (a nuclear marker) was rapidly taken up in both wild-type and Shank2 knockout mice.

The failure of FITC-albumin to be internalized in Shank2 knockout mice after binding to the brush border domain suggests a defect in endocytosis in Shank2 knockout proximal tubular cells. The intravital experiments do not determine how much albumin crosses the GFB and is presented to the apical surface of the proximal tubules. Shank2 knockout mice develop mild proteinuria by 8 weeks of age. While our intravital data do not allow us to directly compare how much albumin crosses the filtration barrier in wild-type versus knockout animals, these experiments suggest that impaired tubular uptake may lead to the development of proteinuria.

Since transmission electron microscopy demonstrates that the structure of the filtration barrier is abnormal in Shank2 knockout mice, proteinuria in the Shank2 KO is likely also due to serum proteins such as albumin leaking through a damaged filtration barrier, bypassing direct endocytosis of albumin by the podocyte. This hypothesis accounts for the findings that Shank2 KO mice have a significant increase in albuminuria compared to wild-type mice (implying that some albumin must make it through the filtration barrier); yet cultured Shank2 KO podocytes have a significant decrease in albumin uptake compared to wild type.

In neurons, endocytosis is a key regulator of multiple processes in neurons including signal transduction, axonal motility, and remodeling of the dendritic spines/PSD (Steketee and Goldberg 2012; Cosker and Segal 2014; Tojima and Kamiguchi 2015). Shank2 plays an important role in organizing proteins at the PSD thereby regulating PSD structure and function. The mechanisms for this regulation include the trafficking of proteins into and out of the plasma membrane. The molecular mechanisms for membrane trafficking includes Shank2 interactions with dynamin, a protein required for pinching off of specific endocytotic vesicle populations (Soda and Ishibe 2013; Sundborger and Hinshaw 2014) and cortactin, a protein involved in actin dynamics (Ammer and Weed 2008).

Previous studies have shown that caveolin-1 is required for albumin endocytosis in podocytes (Dobrinskikh et al. 2014). The present studies evaluated any potential relationship between Shank2 and caveolin-1. When compared to wild-type podocytes, podocytes from Shank2 mice had decreased expression and altered localization of caveolin-1, suggesting that impaired albumin endocytosis in Shank2 knockout podocytes may occur, in part, through dysregulation of caveolin-1 expression. The means by which lack of Shank2 alters caveolin-1 expression remain to be determined. However, since caveolin-1 appears to be mislocalized in Shank2 knockout podocytes, one potential mechanism for the decreased caveolin-1 protein levels seen in the knockout may be due to the fact that in the knockout caveolin-1 is misdirected to the lysosome for degradation.

In summary, Shank2, a dynamic scaffolding protein, is expressed in podocytes and plays an important role in albumin endocytosis. Endocytotic mechanisms in podocytes are believed to be necessary for a number of processes including development, maintenance of the slit diaphragm, and preservation of the GFB (Soda and Ishibe 2013). A detailed understanding of the mechanisms involved in endocytosis in podocytes may ultimately lead to new pathways for treating proteinuric kidney diseases.

## Conflict of Interest

None declared.

## Supporting Information

**Video S1.** Intravital multiphoton movie of albumin trafficking in a wild-type mouse. Images were acquired every 2 sec. Initial images demonstrate tubular autofluorescence. Subsequent images demonstrate tubular uptake of Hoechst dye (blue) which stains nuclei and subsequent injection of FITC-albumin (green) which appears in capillaries and then in the proximal tubular brush border.

**Video S2.** Intravital multiphoton movie of albumin trafficking in a Shank2 knockout mouse. Images were acquired every 2 sec. Initial images demonstrate tubular autofluorescence. Subsequent images demonstrate tubular uptake of Hoechst dye (blue) which stains nuclei and subsequent injection of FITC-albumin (green).

**Video S3.** Intravital multiphoton movie of albumin trafficking in a wild-type mouse 20 min after FITC-albumin injection. FITC-albumin (green) is internalized into renal proximal tubular cells.

**Video S4.** Intravital multiphoton movie of albumin trafficking in a Shank2 knockout mouse 20 min after FITC-albumin injection. There is no internalization of FITC-albumin (green) into renal tubular epithelial cells.
